# 

**Published:** 1853-05

**Authors:** 


### CONTENTS.

ORIGINAL COMMUNICATIONS:— PAGE

ART. I.—Medico-Legal Evidence in Cases of Insanity. By D. Miller, M.D., Chicago, . . . . . . . . . . . . . 1

ART. II.—Remedy for Tape Worm. By J. M'Creary Sudduth, M.D. 6

ART. III.—Biliary Calculi, Obliteration of the Biliary Ducts, Abscess of Liver, &c. By Ira E. Oatman, M.D., Sacramento City, . . . . 7

ART. IV.—Few Years' Obstetrical Practice in the Department of Orcuse, 9

ART. V.—Ascarides. By C. W. Davis, M.D., . . . . . . . 15

ART. VI.—An Inquiry, Critical and Experimental, into the Pathology of Fever. By Professor N. S. Davis, M.D.,—(Continued) . . . 17

SELECTIONS:—

Miasmatic Modifications of Inflammation of the Lungs, . . . 33
Iodide of Sodium in Constitutional Syphilis, . . . . . . . 36
Pneumonia. By Smelfungus, . . . . . . . . . . 37
Case of Portal Phlebitis, . . . . . . . . . . . . 41
Case of Artificial Urethra and Puncture of the Bladder, . . . 42
Anti periodic Properties of the Humulus Lupulus . . . . . . 44
Case of a Female, the mother of seven children, who never menstruated, 45
Death of Prof. Horner, . . . . . . . . . . . . . 47
Case of Constipation, with Vomiting, . . . . . . . . . . 49
Diet as a Remedial Agent in the Treatment of Disease, . . . 51
Remedy for Infantile Asphyxia, . . . . . . . . . . 57
Clinical Examination of the Urine, . . . . . . . . . 58

EDITORIAL:—

A Synodsis or Systematic Catalogue of the Medical Plants of the United States. By A. Clapp, M.D. . . . . . . . . . . 67
Fergusson's System of Practical Surgery, . . . . . . . . . 68
Elements of Health and Principles of Female Hygiene By E. Tilt. M.D. 68
Soleil's Saccharimeter. By W. P. Riddell, . . . . . . . 68
The Druggist's General Receipt Book. By Henry Beasley, . . . . 69
A Clinical Phrase Book &c. By Montgomery Jones, M.D., . . . 69
Lardner's Natural Philosophy, . . . . . . . . . . . 70
American Medical Association, . . . . . . . . . . 70
Cook County Medical Society Proceedings for April . . . . . 79

